# Fibrinolytic Treatment after Transient Ischaemic Attack Caused by Prosthetic Mitral Valve Thrombosis

**DOI:** 10.1155/2016/6809263

**Published:** 2016-05-26

**Authors:** Cornel Koban, Michael Neuß, Grit Tambor, Frank Hölschermann, Christian Butter

**Affiliations:** Immanuel Hospital Bernau Brandenburg Heart Center, Medical School Brandenburg, Ladeburger Straße 17, 16321 Bernau, Germany

## Abstract

Prosthetic valve thrombosis is one of the most severe complications after surgical valve replacement. There are many possible presentations: from asymptomatic to life-threatening complications. We report on a 61-year-old female patient with prosthetic replacement of the aortic and mitral valve in the in-house department of cardiac surgery 3 months ago. The patient was suffering from aphasia during 5 minutes in domesticity. After her presentation in the emergency room, the echocardiographic examination revealed a thrombotic formation of the prosthetic mitral valve. At presentation, the anticoagulation was outside the effective range (INR: 1.7). A successful thrombolytic therapy with the plasminogen activator urokinase was begun with complete resolution of the thrombus.

## 1. Introduction

Prosthetic valve thrombosis is one of the most severe complications after a surgical valve replacement and in most cases caused by insufficient anticoagulation [1]. Phenprocoumon and Coumarine-derivatives are the only approved anticoagulants after a prosthetic valve replacement.

There are many possible presentations of a prosthetic valve thrombosis: from being asymptomatic to syncope, stroke, lung edema, or life-threatening cardiogenic shock [2].

There is a lack of randomized controlled prospective trials in the available literature comparing surgical and thrombolytic therapy in prosthetic valve thrombosis. However, in the literature, many therapy regimens are discussed and still remain controversial [1, 3].

## 2. Case Report

We report on a 61-year-old female patient with prosthetic replacement of the aortic and mitral valve in the in-house department of cardiac surgery 3 months ago due to a combined aortic and mitral valve disease with prevalent valve stenosis. At home, the patient was suffering from aphasia for 5 minutes. There was no evidence of peripheral neurologic deficiency. On hospital admission, an insufficient anticoagulation with an INR of 1.8 was noticeable.

**Figure 1:**
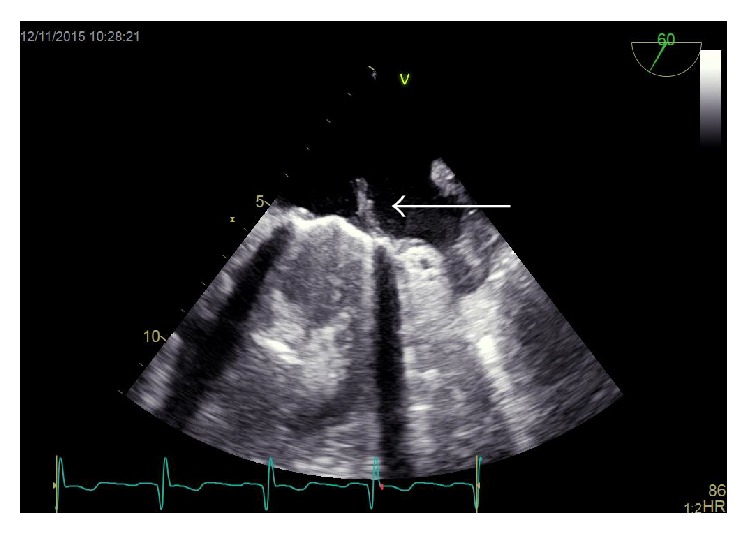
Transesophageal echocardiography of a mitral valve prosthesis. A thrombus originating from the valve ring is visible (arrow).

**Figure 2:**
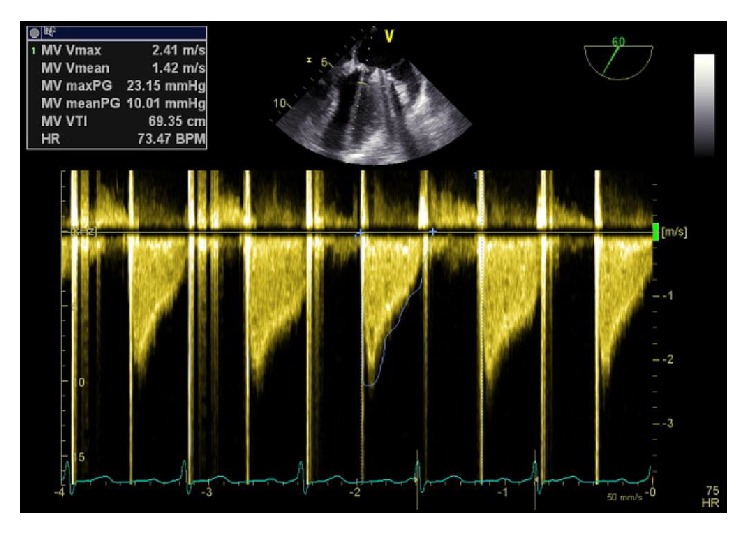
Transesophageal echocardiography of a mitral valve prosthesis. CW-Doppler recording of the transmitral flow shows an increased transvalvular gradient.

There was an adherent thrombotic formation on the prosthetic mitral valve in the echocardiographic examination. Subsequently, a transesophageal echocardiogram was performed which revealed a floating 16 × 6 mm hypodensic structure (previously AML) and a structure of 6 × 2 mm in size (previously PML) on the ring of the mitral valve prosthesis (Figure 1). The mean gradient was moderately to severely elevated to 10 mmHg (Figure 2). The left ventricular function was preserved and there were no thrombotic formations detectable in the left atrial appendix.

Taking the transient neurologic symptoms into account, a CT-scan of the brain was performed. There was neither an evidence of an intracerebral bleeding nor a demarcation of a territorial ischaemia.

After carefully weighting of advantages and disadvantages of a required high dose heparinization for the heart-lung machine (initial bolus heparin 400 IU/kg BW, further dosing to reach an ACT around 500 seconds, terminating with the antagonist protamine necessary), we decided to initiate a first-line low-dose thrombolytic treatment with the plasminogen-activator urokinase on the basis of several years experience (bolus 250.000 IU, maintenance dose 80.000 IU) for 48 hours with concomitant unfractionated heparinization 75 IU/kg/24 h to reach a 1.5-time prolongation of the partial thromboplastin time. Finally, the phenprocoumon therapy was reestablished.

**Figure 3:**
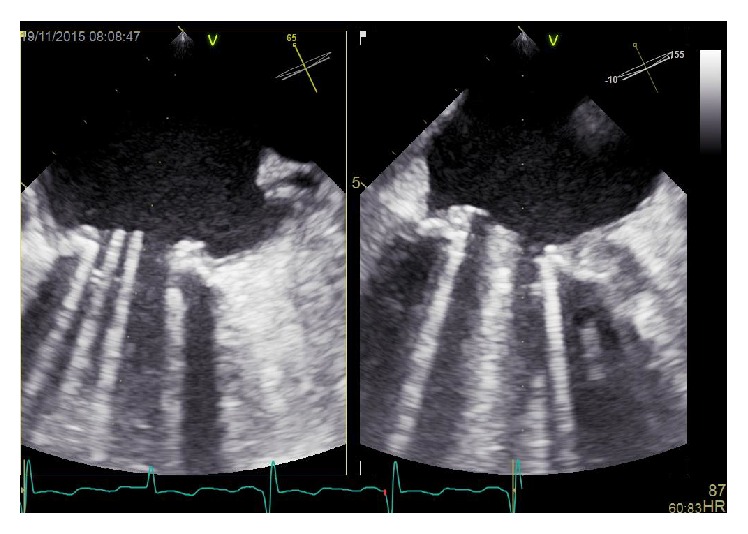
Transesophageal echocardiography of the mitral valve prosthesis after thrombolysis. The thrombus is no longer visible. Normal movement of the disks of the bileaflet valve is visible.

After completion of the thrombolytic therapy, a second transesophageal examination was performed. The floating structures were no longer detected and the mean gradient decreased to 7 mmHg (Figure 3).

Only one of the 7 blood cultures was positive for propionibacterium acnes. This was probably due to a contamination. After one week, new blood cultures were carried out. These showed no germ grove.

After completion of the thrombolytic therapy, the reintroduction of the oral anticoagulants under concomitant unfractionated heparinization was established. An adjuvant ASA medication and a Koagucheck®-Device for INR self-monitoring were prescribed. Discharge from hospital was without subsequent neurological damage.

## 3. Discussion

Valve thrombosis is one of the most common complications after prosthetic valve replacement with an incidence of 0.3 to 1.3 over 100 patient years. The prosthetic mitral valve is affected twice as often as the prosthetic aortic valve. Most of them are nonobstructive [4]. Thromboembolic complications are more frequent and occur in 0.7–6% per patient-year [5–7].

During the first year after prosthetic valve replacement, an incidence of 24% adherent thrombotic material is reported; during the years 2 to 5, the incidence is about 15% and decreases still further [8]. Technological progress with new valve design leads to a more laminar flow with less thrombogenicity according to Virchow's triad [7].

Oral anticoagulants are restricted to phenprocoumon substances that are approved for prosthetic heart valves although direct anticoagulants cause less interactions with comedication and nutrition. The Re-Align-Trial with dabigatran and prosthetic valves was prematurely terminated due to bleeding and thromboembolic complications [9]. The benefit of the adjuvant aspirin therapy remains unclear; there are fewer valve thromboses reported but nevertheless more frequent gastrointestinal bleeding [10].

The transthoracic echocardiographic examination is recommended as the first diagnostic tool of valve thrombosis offering basic information on valve morphology and function. Transesophageal echocardiography is often required to provide more accurate images and to distinguish between pannus and thrombus. A thrombus has usually an echodensity similar to the myocardium while pannus appears more hyperechoic [10].

Since the nineteen-eighties, only radiopaque valve prosthesis was used so that fluoroscopy may also be useful for assessment of valve function by showing restriction of valve leaflet movements in case of prosthetic valve thrombosis [10].

If a cut-off smaller than 5 mm is used for defining small thrombi, the majority of 80% with large thrombi on left-heart sided prosthetic heart valves suffer from serious systemic embolization [4, 11, 12].

Up to the 1990s, surgery was the preferred treatment in the case of thrombosis of prosthetic heart valves. Depending on the preoperative state of the patient, the mortality rate of surgical thrombectomy or valve replacement was approximately 69% [13, 14].

During recent years, the thrombolytic therapy is increasingly performed to spare the patient surgery. The TROIA-Trial compared a variety of thrombolytic regimes. Different authors recorded a successful thrombolysis in 73–83% [12, 15–17]. Possible complications might be reversible systemic embolization in 10−18% [15, 18, 19]. Fatal outcomes are reported in 2.8–11.8% [15, 20–22].

Low-dose fibrinolytic prolonged regimens with tPA should lower complication rates further. For this purpose, the SAFE-PVT-Trial (Surgery versus Fibrinolytic Therapy for Left-Sided Prosthetic Heart Valve Thrombosis, NCT01641549) is currently in progress. A second ongoing study compares surgery and thrombolytic therapy in patients with obstructive prosthetic valve thrombosis (NCT02243839). There is no consensus in the literature concerning the best fibrinolytic regimen. A simple therapeutic strategy can be proposed with two types of protocols. In patients with haemodynamic instability, “rescue” fibrinolysis should be preferred, using a “short protocol.” Clinical stable patients should be included in a “long protocol.”

## 4. Conclusion

The clinical outcome of small thrombi smaller than 5 mm is mostly benign and requires an adaption of the oral anticoagulants. Patients with larger nonobstructive thrombi develop more often further complications and require a more aggressive therapeutic regimen. Bridging the gap in the anticoagulation with unfractionated heparin until the reachievement of the required INR-range is one of the early goals. The adjuvant aspirin therapy leads to fewer valve thromboses; however, more often gastrointestinal bleeding is to be noticed. In recent years, a first-line fibrinolytic therapy is performed more often, but more kinds of reversible systemic embolization are recorded. If primary therapy, based on thrombolytic treatment, fails, therapists should consider resurgery, but taking the risks, as outlined, into consideration.

## References

[1] Lengyel M., Vándor L. (2001). The role of thrombolysis in the management of left-sided prosthetic valve thrombosis: a study of 85 cases diagnosed by transesophageal echocardiography. *Journal of Heart Valve Disease*.

[2] Lengyel M., Vegh G., Vandor L. (1999). Thrombolysis is superior to heparin for non-obstructive mitral mechanical valve thrombosis. *Journal of Heart Valve Disease*.

[3] Bollag L., Attenhofer Jost C. H., Vogt P. R. (2001). Symptomatic mechanical heart valve thrombosis: high morbidity and mortality despite successful treatment options. *Swiss Medical Weekly*.

[4] Laplace G., Lafitte S., Labèque J.-N. (2004). Clinical significance of early thrombosis after prosthetic mitral valve replacement: a postoperative monocentric study of 680 patients. *Journal of the American College of Cardiology*.

[5] Cannegieter S. C., Rosendaal F. R., Briët E. (1994). Thromboembolic and bleeding complications in patients with mechanical heart valve prostheses. *Circulation*.

[6] Horstkotte D., Burckhardt D. (1995). Prosthetic valve thrombosis. *Journal of Heart Valve Disease*.

[7] Roudaut R., Serri K., Lafitte S. (2007). Thrombosis of prosthetic heart valves: diagnosis and therapeutic considerations. *Heart*.

[8] Deviri E., Sareli P., Wisenbaugh T., Cronje S. L. (1991). Obstruction of mechanical heart valve prostheses: clinical aspects and surgical management. *Journal of the American College of Cardiology*.

[9] Eikelboom J. W., Connolly S. J., Brueckmann M. (2013). Dabigatran versus warfarin in patients with mechanical heart valves. *The New England Journal of Medicine*.

[10] Laffort P., Roudaut R., Roques X. (2000). Early and long-term (one-year) effects of the association of aspirin and oral anticoagulant on thrombi and morbidity after replacement of the mitral valve with the st. jude medical prosthesis: a clinical and transesophageal echocardiographic study. *Journal of the American College of Cardiology*.

[11] Gueret P., Vignon P., Fournier P. (1995). Transesophageal echocardiography for the diagnosis and management of nonobstructive thrombosis of mechanical mitral valve prosthesis. *Circulation*.

[12] Biteker M., Altun I., Basaran O., Dogan V., Yildirim B., Ergun G. (2015). Treatment of prosthetic valve thrombosis: current evidence and future directions. *Journal of Clinical Medicine Research*.

[13] Martinell J., Jiménez A., Rábago G., Artiz V., Fraile J., Farré J. (1991). Mechanical cardiac valve thrombosis. Is thrombectomy justified?. *Circulation*.

[14] Mehra S., Movahed A., Espinoza C., Marcu C. B. (2015). Horseshoe thrombus in a patient with mechanical prosthetic mitral valve: a case report and review of literature. *World Journal of Clinical Cases*.

[15] Lengyel M., Fuster V., Keltai M. (1997). Guidelines for management of left-sided prosthetic valve thrombosis: a role for thrombolytic therapy. *Journal of the American College of Cardiology*.

[16] Nagy A., Dénes M., Lengyel M. (2009). Predictors of the outcome of thrombolytic therapy in prosthetic mitral valve thrombosis: a study of 62 events. *Journal of Heart Valve Disease*.

[17] Özkan M., Gündüz S., Biteker M. (2013). Comparison of different TEE-guided thrombolytic regimens for prosthetic valve thrombosis: the TROIA trial. *JACC: Cardiovascular Imaging*.

[18] Fitzpatrick M. A., Ikram H., Ilsley C. (1988). Acute thrombotic obstruction of a mitral valve prosthesis: the role of thrombolytic therapy. *Australian and New Zealand Journal of Medicine*.

[19] Graver L. M., Gelber P. M., Tyras D. H. (1988). The risks and benefits of thrombolytic therapy in acute aortic and mitral prosthetic valve dysfunction: report of a case and review of the literature. *Annals of Thoracic Surgery*.

[20] Özkan M., Kaymaz C., Kirma C. (2000). Intravenous thrombolytic treatment of mechanical prosthetic valve thrombosis: a study using serial transesophageal echocardiography. *Journal of the American College of Cardiology*.

[21] Ermis N., Atalay H., Altay H., Bilgi M., Binici S., Sezgin A. T. (2011). Comparison of fibrinolytic versus surgical therapy in the treatment of obstructive prosthetic valve thrombosis: a single-center experience. *Heart Surgery Forum*.

[22] Gazi E., Altun B., Temiz A., Colkesen Y. (2013). Successful thrombolytic treatment of prosthetic mitral valve thrombosis. *BMJ Case Reports*.

